# Diverse yeast antiviral systems prevent lethal pathogenesis caused by the L-A mycovirus

**DOI:** 10.1073/pnas.2208695120

**Published:** 2023-03-08

**Authors:** Sabrina Chau, Jie Gao, Annette J. Diao, Shi Bo Cao, Amirahmad Azhieh, Alan R. Davidson, Marc D. Meneghini

**Affiliations:** ^a^Department of Molecular Genetics, University of Toronto, Toronto, ON M5G 1M1, Canada; ^b^Department of Biochemistry, University of Toronto, Toronto, ON M5G 1M1, Canada

**Keywords:** yeast, dsRNA L-A mycovirus, antiviral factors, viral pathogenesis, proteotoxic stress

## Abstract

Recent studies show that antiviral systems are remarkably conserved from bacteria to mammals, demonstrating that unique insights into these systems can be gained by studying microbial organisms. Unlike in bacteria, however, where phage infection can be lethal, no cytotoxic viral consequence is known in the budding yeast *Saccharomyces cerevisiae* even though it is chronically infected with a double-stranded RNA mycovirus called L-A. This remains the case despite the previous identification of conserved antiviral systems that limit L-A replication. Here, we show that these systems collaborate to prevent rampant L-A replication, which causes lethality in cells grown at high temperature. Exploiting this discovery, we use an overexpression screen to identify antiviral functions for the yeast homologs of polyA-binding protein (PABPC1) and the La-domain containing protein Larp1, which are both involved in viral innate immunity in humans. Using a complementary loss of function approach, we identify new antiviral functions for the conserved RNA exonucleases *REX2* and *MYG1*; the SAGA and PAF1 chromatin regulatory complexes; and *HSF1*, the master transcriptional regulator of the proteostatic stress response. Through investigation of these antiviral systems, we show that L-A pathogenesis is associated with an activated proteostatic stress response and the accumulation of cytotoxic protein aggregates. These findings identify proteotoxic stress as an underlying cause of L-A pathogenesis and further advance yeast as a powerful model system for the discovery and characterization of conserved antiviral systems.

## Significance

The budding yeast *Saccharomyces cerevisiae* is chronically infected with a double-stranded RNA virus called L-A that has had no known fitness consequence. Controverting its presumed harmless nature, we show here that L-A causes proteotoxic stress and conditional lethality in cells lacking parallel-acting viral attenuation pathways. Taking advantage of the genetic tools available in budding yeast, we identify several highly conserved proteins to play a role in antiviral defense. Some of these have been recently identified in humans to be involved in viral innate immunity, thus highlighting the potential of budding yeast as a model organism to identify and investigate new antiviral systems.

All laboratory strains and most environmental isolates of the budding yeast *S. cerevisiae* are infected with a double-stranded RNA (dsRNA) virus called L-A (1, 2). L-A belongs to the broadly dispersed *Totiviridae* family of endogenous dsRNA viruses. Like all viruses of this family, the L-A dsRNA genome is packaged within a virion that shields it from host-mediated digestion. Holes in the virion permit the extrusion of RNA transcripts into the cytosol that encode the capsid protein, Gag, which comprises most of the particle. The L-A transcript also encodes a Gag-pol fusion protein, produced at much lower levels than the Gag protein, that possesses RNA-dependent RNA polymerase activity. Each virion contains a Gag-pol protein, which accounts for L-A replication and transcription within the particle. Encapsidation of the viral transcripts within nascent particles and synthesis of the negative RNA strand by Gag-pol to form the dsRNA genome completes the L-A replication cycle (2). To produce these proteins, L-A employs features typical of RNA viruses found in humans, including a “cap-snatching” mechanism that furnishes L-A transcripts with a 5′-methyl cap and a ribosomal frameshifting mechanism to produce Gag and Gag-pol fusion proteins from a single transcript (3, 4).

Recent studies of bacterial antiviral systems have shown that they share remarkable evolutionary conservation with humans, revealing the potential of microbial organisms to provide new insights into viral innate immunity (5–11). Indeed, early studies involving L-A led to the discovery of two antiviral systems that have subsequently been shown to contribute to innate immunity against diverse RNA viruses in mammals (12–17). The first of these antiviral systems involves the *SKI2*, *3*, and *8* genes, which encode subunits of a conserved ribosome-associated complex that opposes the translation of transcripts that lack poly(A) tails like those encoded by L-A (18–23). A separate pathway of L-A attenuation occurs through Xrn1 (also known as *SKI1*), a 5′-3′ exoribonuclease that degrades uncapped mRNAs (24–26).

We recently found that the mitochondrial DNA/RNA endonuclease Nuc1 represses the accumulation of L-A in sporulating cells, representing a new yeast antiviral pathway (27). Nuc1 is a homolog of endonuclease G (EndoG) found in all eukaryotes and many prokaryotes and is most known for its role in promoting genome fragmentation during mammalian programmed cell death, a prominent last-resort mechanism of viral defense (28, 29). Intriguingly, programmed cell death is intrinsic to yeast sporulation, and Nuc1 fragments the DNA from dying meiotic products during this process in addition to its role in attenuating L-A viral levels that are inherited by the surviving spores (27, 30, 31).

Despite the ubiquitous presence of L-A in lab strains, there has been no fitness consequence attributed to it and thus L-A is largely regarded as a harmless commensal. Here, we show that L-A infection is in fact deadly for yeast and that it must be actively attenuated through viral innate immunity to preserve viability. Specifically, in strains lacking parallel-acting *NUC1* and *SKI* antiviral pathways, L-A copy number is massively increased, leading to lethality at high temperature.

We reasoned that further characterization of L-A and the factors that maintain its replication at a low level could reveal new antiviral systems. Identifying conditions that lead to L-A pathogenesis allowed us to use bioinformatic and forward genetic screening approaches to discover new antiviral genes. Using a screen for overexpressed genes that suppress *nuc1∆ ski3∆* conditional lethality, we identify antiviral functions for the yeast homologs of poly(A)-binding protein (PABPC1) and the La-domain containing protein Larp1, which are both involved in viral innate immunity in humans (32, 33). Moreover, loss of function genetic studies identified twelve new antiviral genes. Among these are the highly conserved SAGA transcriptional coactivator complex and several RNA exonucleases including *REX2* and *MYG1*, both of which have distinct but poorly characterized human and bacterial homologs (34–37).

Finally, we characterize L-A pathogenesis using cell biological methods and find that high viral load causes proteostatic stress. As high temperature is well known to exacerbate proteostatic stress, these observations suggest that catastrophic proteostatic stress is the cause of L-A–induced lethality. Consistent with this hypothesis, we show that *nuc1∆ ski3∆* mutants exhibit L-A-dependent sensitivity to azetidine-2-carboxylic acid (AZC), a proline analog known to cause proteostatic stress (38). Further, we demonstrate an antiviral function for *HSF1*, a conserved transcription factor that senses and directs the response to proteostatic stress. Interestingly, human Hsf1 also plays important roles in the replication and/or pathogenicity of diverse viruses including HIV, SARS-Cov-2, and dengue virus, though the mechanisms are unclear (39). These findings provide novel examples of innate immune conservation from microbes to humans and further highlight yeast as a powerful model system for the discovery of new antiviral systems.

## Results

### The NUC1, SKI, and XRN1 Yeast Antiviral Systems Collaborate to Prevent L-A Pathogenesis.

**Fig. 1.**
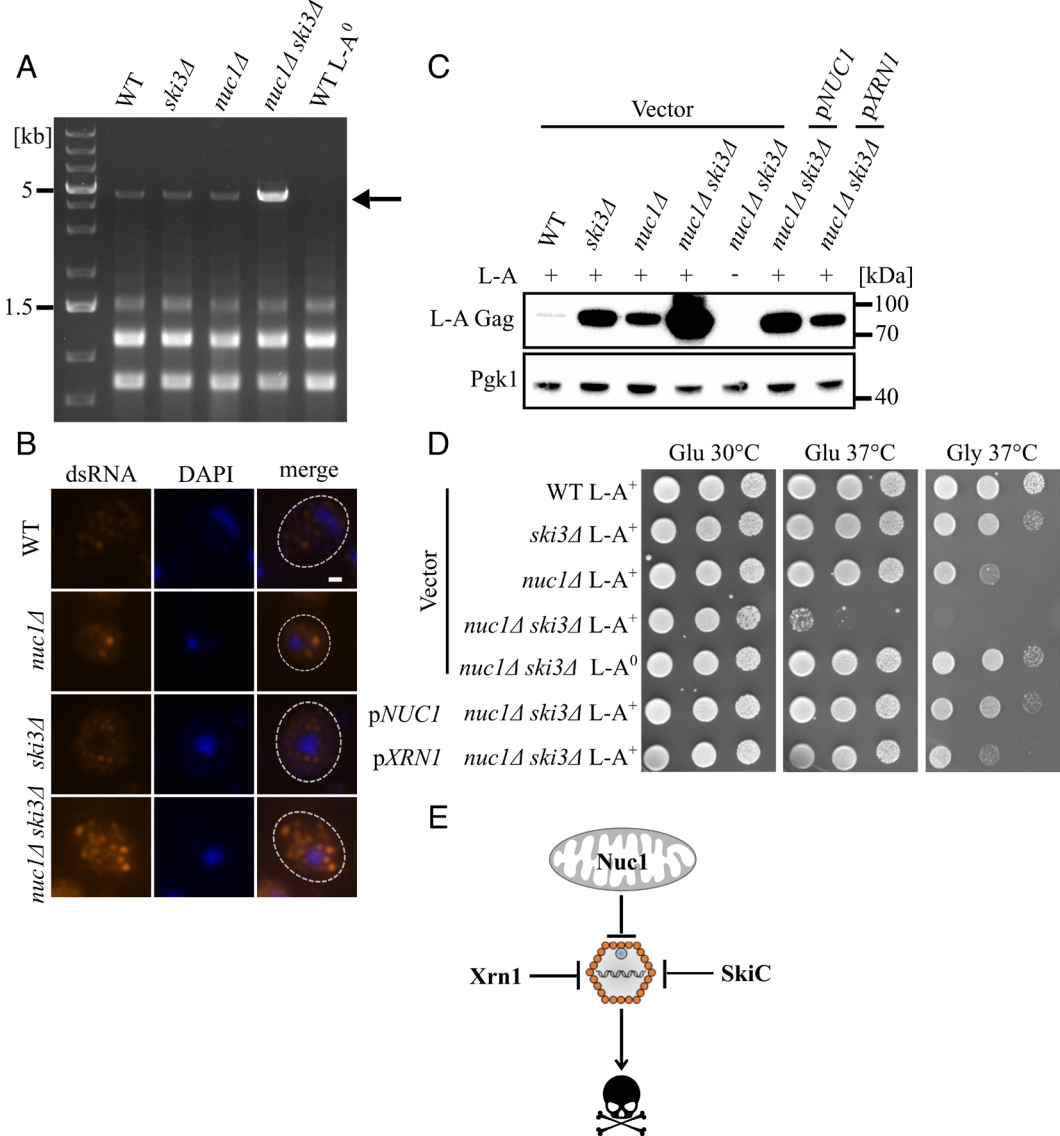
L-A attenuation protects yeast from lethal pathogenesis. (*A*) An ethidium bromide-stained gel of total RNA prepared from the indicated strains is shown, with the 4.6 kb L-A dsRNA band indicated with an arrow. (*B*) Immunofluorescence was used to visualize L-A dsRNA (orange) in cells of the indicated genotypes. These strains were cured of the weakly abundant L-BC dsRNA virus to eliminate background staining (*Method Details*). DAPI staining of DNA is in blue. (Scale bar, 1 μm.) (*C*) Western blotting of L-A Gag and 3-phosphoglycerate kinase (Pgk1) protein levels in the indicated strains is shown. Molecular weight markers are indicated on the right. (*D*) Spot test growth assays of the strains from 1C is shown. Strains were spotted on -Leu media containing either glucose or glycerol and grown at the indicated temperatures. (*E*) The mitochondrial protein Nuc1 collaborate with the cytosolic proteins Xrn1 and SkiC to regulate L-A protein level and ensure cell fitness.

Our previous studies of *NUC1* were focused on meiotic cells (27). To investigate *NUC1* antiviral function in vegetatively growing yeast, we examined L-A copy number in mitotic haploid cells in the reference BY4742 strain background. We observed the levels of L-A dsRNA using ethidium bromide staining of electrophoresed RNA and found that a *nuc1∆ ski3∆* double mutant showed a large increase in L-A dsRNA (Fig. 1*A*). We corroborated these findings using immunofluorescence microscopy with a dsRNA antibody used to detect replicating RNA viruses (40, 41). These images showed that L-A dsRNA accumulated in foci, reminiscent of “viral factory” sites of viral replication observed in human cells (Fig. 1*B* and *SI Appendix*, Fig. S1) (42). Consistent with previous findings in other strain backgrounds (24, 27, 43), western blotting showed that Gag protein levels were elevated in the *nuc1∆* and *ski3∆* mutants (Fig. 1*C*). Furthermore, we showed that a *nuc1∆ ski3∆* double mutant accumulated massively elevated Gag levels (Fig. 1*C*). These data show that *NUC1* and *SKI3* participate in separate antiviral pathways and that loss of both pathways results in a greatly increased L-A viral load.

To determine if high L-A viral load affects cell fitness, we examined yeast growth using spot test growth assays. Subtle growth defects of *nuc1∆* and *ski3∆* single mutants were observed at 37 °C when cells were grown with glycerol rather than glucose as the carbon source, a condition under which yeast relies on mitochondrial respiration (Fig. 1*D*). Remarkably, although *nuc1∆ ski3∆* double mutants grew normally at 30 °C, they exhibited conditional lethality at 37 °C regardless of the carbon source (Fig. 1*D*). As expected, viability at high temperature was restored to a *nuc1∆ ski3∆* double mutant by a *NUC1*-expressing plasmid which elicited a corresponding decrease in Gag levels (Fig. 1 *C* and *D*). To confirm that the growth defect of the *nuc1∆ ski3∆* double mutant was caused by L-A, we constructed an isogenic strain cured of L-A (L-A^0^) and assayed its growth at high temperature. We found that the growth defect was completely alleviated, implying that the conditional lethality was a result of unrestricted L-A replication (Fig. 1*D*). To assess the effects of high L-A copy number on cell fitness under optimal growth conditions, we measured proliferation rates in liquid culture. These studies revealed a reduced growth rate in *nuc1∆ ski3∆* double mutants compared to wild type at 30 °C that was reversed in L-A^0^ strains, demonstrating that high L-A load is detrimental for fitness even in nonstressed cells (*SI Appendix*, Fig. S2).

To further characterize how *NUC1* interacts with known antiviral pathways, we tested its relationship with *XRN1*. We found that a *nuc1∆ xrn1∆* double mutant accumulated greatly elevated levels of Gag compared with either single mutant and exhibited L-A–dependent conditional lethality at high temperature (*SI Appendix*, Fig. S3 *A* and *B*), suggesting that *NUC1* and *XRN1* act in parallel pathways to attenuate L-A. Reflecting their key nonredundant roles in bulk mRNA regulation, an *xrn1∆ ski3∆* double mutant is inviable, even in strains lacking L-A (44). To determine whether *XRN1* represents an antiviral system independent of both *NUC1* and *SKI3*, we used a high-copy plasmid to overexpress *XRN1* in a *nuc1∆ ski3∆* double mutant. Indeed, we observed a substantial decrease in Gag levels and suppression of the *nuc1∆ ski3∆* conditional lethality using plasmid-driven *XRN1* overexpression (Fig. 1 *C* and *D*). We conclude that Nuc1, Ski3, and Xrn1 convergently oppose L-A replication and that massively increased L-A viral load in *nuc1∆ ski3∆* or *nuc1∆ xrn1∆* mutants caused lethal pathogenesis at high temperature (Fig. 1*E*).

### A BioinformaticBased Genetic Screen Identifies New Antiviral Factors.

The L-A–dependent conditional lethality of *nuc1∆ ski3∆* double mutants raised the possibility that other antiviral factors could be identified through combinatorial mutant studies. To identify new candidate antiviral factors, we searched a curated genetic interaction database for gene deletions that caused a synthetic growth defect when combined with *nuc1∆* in at least two high-throughput screening studies (45). In addition to the expected presence of *XRN1* and *SKI* deletions in this data set, we found sixteen additional genes. We used genetic crossing to make triple mutants combining deletions of each of these sixteen genes with *nuc1∆ ski3∆* and confirmed six that caused severe growth defects (Table 1). We determined that the synthetic growth phenotypes caused by each of these genes were reversed in L-A^0^ strains, suggesting that they encode antiviral proteins (Table 1). We describe confirmation of several of these screen hits as new antiviral factors below.

**Table 1.** Identification of new candidate antiviral factors using a bioinformatic approach

	Synthetic growth phenotypes with				
Mutation	***nuc1*Δ**	***ski3*Δ**	***nuc1*Δ *ski3*Δ**	**L-A^0^ *nuc1*Δ *ski3*Δ**	Gene description
***rex2*Δ**	**Lethal (37 °C)**	**None**	**Lethal**	**None**	**3′-5′ RNA exonuclease**
***myg1*Δ**	**Sick (37 °C)**	**None**	**Lethal (35 °C)**	**None**	**3′-5′ RNA exonuclease**
***rnh70*Δ**	**Sick (37 °C)**	**None**	**Lethal**	**None**	**3′-5′ RNA exonuclease**
***spt3*Δ**	**Lethal (37 °C)**	**None**	**Lethal**	**None**	**SAGA complex subunit**
*spt8*Δ	Lethal (37 °C)	None	Lethal	n.d.	SAGA complex subunit
*gcn5*Δ	Lethal (37 °C)	None	Very sick	n.d.	SAGA complex subunit
*sgf73*Δ	Lethal (37 °C)	None	Very sick	n.d.	SAGA complex subunit
***maf1*Δ**	**Sick (glycerol)**	**None**	**Lethal (glycerol)**	**None**	**RNA PolIII regulator**
***cdc73*Δ**	**Lethal (37 °C)**	**None**	**Lethal**	**None**	**Paf1 complex subunit**
*rtf1*Δ	Lethal (37 °C)	None	Very sick	None	Paf1 complex subunit
*leo1*Δ	Lethal (37 °C)	None	Very sick	None	Paf1 complex subunit

Strains with gene deletions of candidate antiviral factors combined with *nuc1∆*, *ski3∆*, or *nuc1∆ ski3∆* were engineered using tetrad dissections. Listed are the positive hits with their according synthetic growth phenotypes and L-A dependency. The original six genes identified in the bioinformatics screen are bolded. Unless indicated, growth conditions are 30 °C with glucose as the carbon source (n.d. = not determined).

**Fig. 2.**
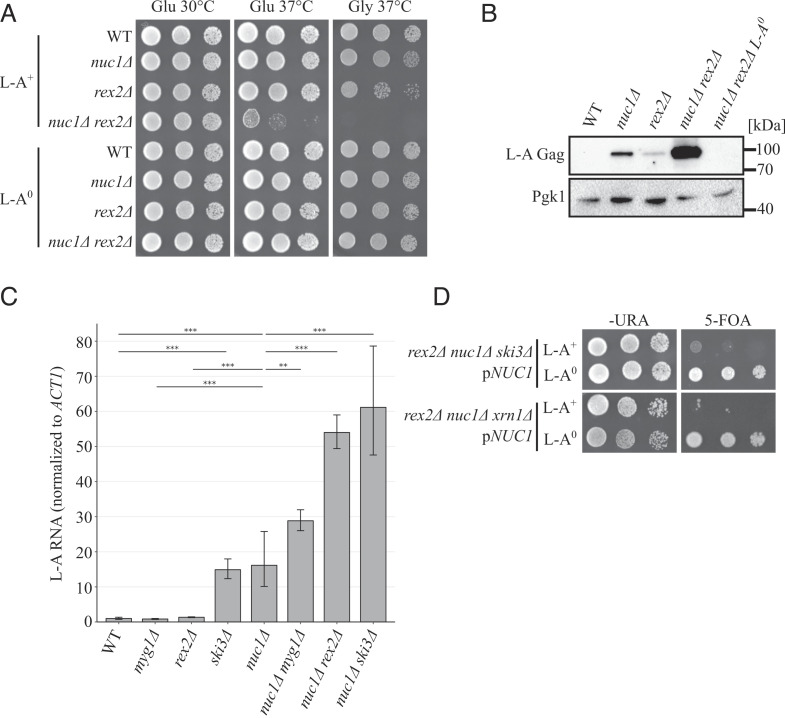
New antiviral factors are identified by exploiting L-A pathogenesis. (*A*) Spot analysis of strains defective in *NUC1* and *REX2* is shown. Strains were spotted on SC media containing either glucose or glycerol and grown at the indicated temperature. (*B*) Western blotting of L-A Gag and Pgk1 protein levels of strains in Fig. 2*A*. Molecular weight markers are indicated on the right. (*C*) L-A RNA was quantified by qPCR and normalized to endogenous *ACT1* RNA. Mean RNA level and SD is shown. n = 5. *P < 0.05, **P < 0.01, ***P < 0.001 (unpaired Student’s *t* test). (*D*) Spot analysis of strains defective in three parallel antiviral pathways containing plasmid expressing *NUC1* is shown. Strains are spotted on -URA media or synthetic complete (SC) media supplemented with 0.1% 5-fluoroorotic acid (5-FOA).

One gene identified in our screen, *REX2*, encodes a 3′-5′ RNA exonuclease conserved from bacteria to humans (35). Both Rex2 and its human homolog REXO2 localize to the mitochondria and contain an EXOIII domain widely found in prokaryotic and eukaryotic proteins, including the interferon-stimulated antiviral protein ISG20 (36, 37, 46–48). We found that a *rex2∆ nuc1∆* double-mutant strain accumulated greatly increased levels of Gag compared to either single mutant and exhibited L-A–dependent growth defects, including lethality at high temperature (Fig. 2 *A* and *B*, and *SI Appendix*, Fig. S2). A *rex2*∆ single-mutant strain exhibited a slight increase in Gag levels, though this effect was marginal (Fig. 2*B*). To rigorously scrutinize the consequences of *rex2∆* for L-A copy number, we quantified L-A RNA using RT-qPCR. These measurements confirmed that *rex2∆ nuc1∆* strains accumulate greatly increased levels of L-A, though they also revealed that a *rex2∆* single mutant did not accumulate increased L-A RNA (Fig. 2*C*). These findings suggest Rex2’s antiviral role is apparent only in the absence of *NUC1* function. Remarkably, *nuc1∆ ski3∆ rex2∆* and *nuc1∆ xrn1∆ rex2∆* triple mutants were inviable under all growth conditions, and these defects were reversed in L-A^0^ strains (Fig. 2*D*). These findings demonstrate the severe pathogenic potential of the L-A mycovirus and identify a new antiviral role for a highly conserved mitochondrially localized RNA exonuclease.

Another gene identified in our screen was *MYG1*, the yeast homolog of human MelanocYte proliferation Gene 1, a 3′-5′ RNA exonuclease which has homologs in all taxa (34). Mutant strains combining *myg1∆* and *nuc1∆* exhibited large increases in Gag protein and L-A RNA compared to the single mutants and displayed severe L-A-dependent growth defects at high temperature and in liquid culture (Fig. 2*C* and *SI Appendix*, Figs. S2, S4 *A* and *C*). Like with *rex2∆*, a *myg1∆* single mutant strain showed little increase in Gag level and no change in L-A RNA (Fig. 2*C* and *SI Appendix*, Fig. S4*C*). We were able to recover *nuc1∆ ski3∆ myg1∆* triple mutants, though they were extremely slow growing at 30 °C and accumulated even higher levels of Gag (*SI Appendix*, Fig. S4 *A* and *C*). These growth defects were also reversed in L-A^0^ strains (*SI Appendix*, Fig. S4*A*). *MYG1* thus represents a new antiviral factor, acting in parallel to both *NUC1* and the *SKI* complex.

Mutations leading to overexpression of human MYG1 are associated with the autoimmune disorder vitiligo, suggesting that MYG1 may play some role in human innate immunity (49, 50). We explored this possibility using a plasmid expressing human MYG1 under the control of a constitutive yeast promoter (34) and found that human MYG1 rescued the conditional growth defect of a *nuc1∆ myg1∆* mutant (*SI Appendix*, Fig. S4*D*). These findings show that the antiviral function of yeast *MYG1* can be accomplished by human MYG1, suggesting a potential antiviral function for MYG1 in humans.

Another gene category identified using our bioinformatic screen was gene expression. *CDC73* and *SPT3* encode subunits of the conserved chromatin associated complexes PAF1 and SAGA, respectively. Both *cdc73∆* and *spt3∆* caused L-A-dependent lethality when combined with *nuc1∆ ski3∆* (Table 1). Because SAGA (Spt-Ada-Gcn5-Acetyltransferase) has been shown to upregulate antiviral gene expression in the chestnut blight fungus *Cryphonectria parasitica*, we investigated this complex further (51). A *spt3∆ nuc1∆* double-mutant strain accumulated increased levels of Gag and exhibited L-A–dependent lethality at high temperature (*SI Appendix*, Fig. S4 *B* and *C*). SAGA is a large protein complex and we confirmed that deletions in several other SAGA subunit-encoding genes had the same phenotypic consequences as *spt3∆* (*SI Appendix*, Table S1 and S4*C*). Together with the findings from *C. parasitica*, these results suggest that the SAGA complex controls antiviral gene expression in diverse fungal species.

### High Copy Suppression Screening Identifies Yeast Antiviral Factors that Are Also Antiviral in Humans.

**Fig. 3.**
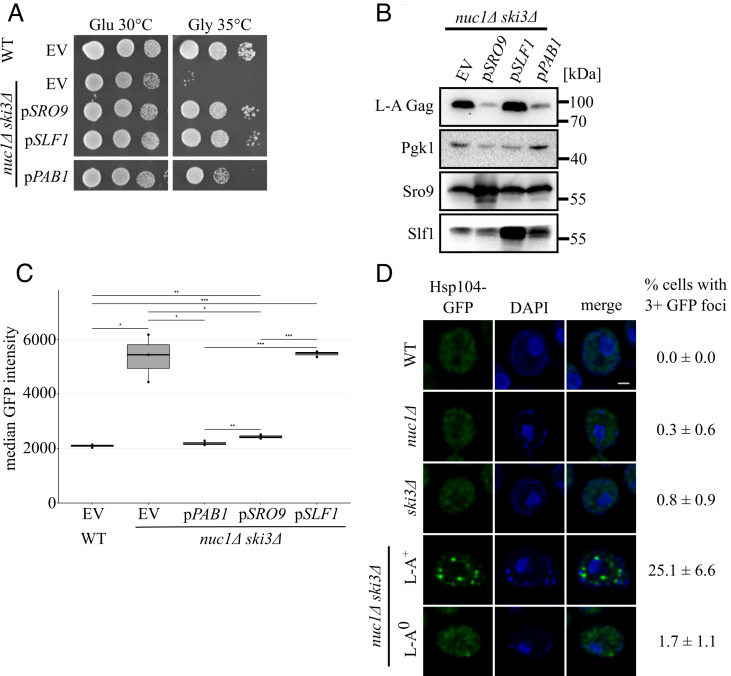
Overexpression of translation control factors alleviate L-A pathogenesis. (*A*) Spot test growth assays of the high copy suppressors *SRO9*, *SLF1*, and *PAB1* are shown. Strains were spotted on –LEU media containing either glucose or glycerol and grown at the indicated temperatures. (*B*) Western blotting for L-A Gag, Pgk1, Sro9, and Slf1 protein levels in the strains from 3A. Molecular weight markers are indicated on the right. (*C*) Flow cytometry was used to measure HSE-GFP expression in the indicated strains (n = 3). First and third quartiles are marked by the grey boxes. Median GFP intensity is marked by the black bars within. *P < 0.05, **P < 0.01, ***P < 0.001 (unpaired Student’s *t* test). (*D*) Fluorescence microscopy of Hsp104-GFP in indicated strains. Cells were stained with DAPI to visualize nuclei. (Scale bar, 1 μm.) Percentage of cells with 3+ GFP foci is shown on the right. n = 3. 75 to 140 cells were counted for each replicate.

Since *XRN1* overexpression suppressed the growth defects of a *nuc1∆ ski3∆* strain, we hypothesized that overexpression of other antiviral factors would produce a similar effect, which could be used as a screen to identify new antiviral systems. We employed a high-copy plasmid suppression screen to detect genes whose overexpression alleviated the conditional lethality of a *nuc1Δ ski3Δ* strain (*Method Details*). Using this screen, we identified *SRO9*, *SLF1*, and *PAB1* as high-copy suppressors of *nuc1∆ ski3∆*, all of which encode ribosome-associated RNA binding proteins (Fig. 3*A*) (52, 53). Sro9 and Slf1 are paralogous lupus-autoantigen (La) domain containing proteins broadly found in eukaryotes. Notably, their human homolog, Larp1, was recently identified in screens for proteins bound to the SARS-Cov-2 plus strand ssRNA or nucleocapsid (32, 54). Larp1 was a major focus in one of these studies and was shown to attenuate SARS-Cov-2 replication in human cells, though its mechanism is not known (32). *PAB1* encodes the highly conserved PolyA-Binding Protein, which is a common target of viral inhibition in humans through diverse mechanisms (33).

We found that overexpression of *PAB1* or *SRO9* markedly reduced Gag levels in a *nuc1∆ ski3∆* mutant, explaining their rescuing phenotypes (Fig. 3*B*). Curiously, even though *SLF1* overexpression rescued the *nuc1∆ ski3∆* growth defect just as well as *SRO9*, it did not lead to any reduction in Gag levels (Fig. 3 *A* and *B*). These findings suggest that *PAB1* and *SRO9* rescue cells by suppressing L-A replication and that *SLF1* protects cells from the pathogenic consequences of elevated viral replication.

### High L-A Copy Number Leads to Cytotoxic Proteostatic Stress.

To gain insight into the divergent mechanisms of Sro9 and Slf1 antiviral activities, we considered what the physiological consequences of L-A pathogenesis could be and how *SRO9*/*SLF1* might differentially impact them. We noted a previous study in which deletions of *NUC1* or of *SKI*-complex genes led to weak induction of a GFP reporter gene controlled by Hsf1 (55), a conserved transcription factor that senses proteostatic stress and activates the gene expression response (56–58). Using flow cytometry with this reporter (HSE-GFP), we confirmed these results and determined that a *nuc1∆ ski3∆* double mutant caused synergistic and L-A–dependent activation of HSE-GFP (Fig. 3*C* and *SI Appendix*, Fig. S5). We hypothesized that the massive production of Gag observed in *nuc1∆ ski3∆* mutants accounted for this proteostatic stress response. Supporting this, HSE-GFP activation of a *nuc1∆ ski3∆* double mutant was reverted by *PAB1* or *SRO9* overexpression, mirroring these genes consequences for Gag accumulation (Fig. 3 *B* and *C*). Notably, overexpression of the *SRO9* paralog *SLF1* did not prevent HSE-GFP activation. Evolutionary divergence of the paralogous *SRO9* and *SLF1* genes has thus resulted in different antiviral mechanisms, with *SRO9* suppressing viral protein accumulation and associated proteostatic stress and *SLF1* seemingly protecting cells from the toxic consequences of viral-induced proteostatic stress.

Proteostatic stress is often associated with the accumulation of cytotoxic protein aggregates that can be visualized using GFP fused to the protein disaggregase Hsp104, a direct target of Hsf1 transcriptional activation that is known to co-localize with protein aggregates (59, 60). To further explore the proteostatic defects associated with L-A pathogenesis, we used fluorescence microscopy to visualize Hsp104-GFP foci in a variety of strains. As expected, wild-type cells grown at 30 °C rarely accumulated observable Hsp104-GFP foci. While *nuc1∆* and *ski3∆* single mutants resembled wild type, strikingly, a *nuc1∆ ski3∆* double mutant exhibited more than 25% of cells with three or more Hsp104-GFP foci (Fig. 3*D*). As with all other phenotypes we have observed for *nuc1∆ ski3∆*, the accumulation of Hsp104-GFP foci was dependent on the presence of L-A (Fig. 3*D*). These findings show that high viral load caused by deletion of *NUC1* and *SKI3* led to the accumulation of Hsp104-GFP foci indicative of cytotoxic protein aggregation.

**Fig. 4.**
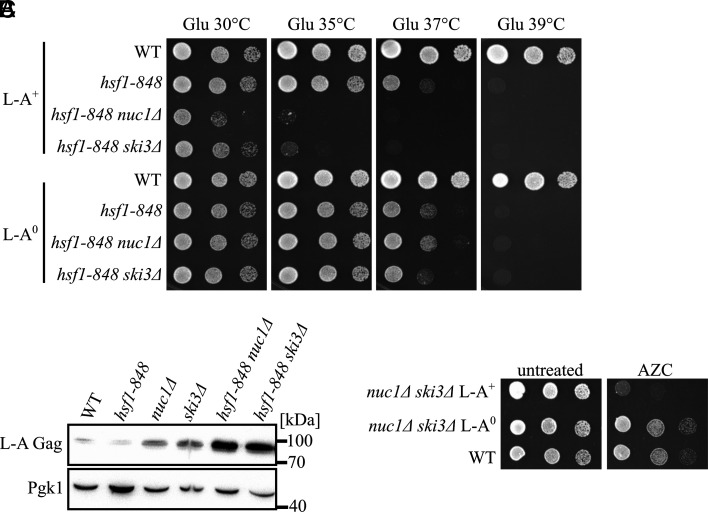
The heat shock response suppresses L-A pathogenesis. (*A*) Spot analysis of strains defective in *HSF1*, *NUC1*, and *SKI3* with or without L-A is shown. Strains were spotted on SC media containing glucose and grown at the indicated temperature. (*B*) Western blotting for L-A Gag and Pgk1 protein levels of indicated strains. Molecular weight markers are indicated on the right. (*C*) Spot analysis of strains treated with the proteotoxic proline analog, azetidine-2-carboxylic acid (AZC), is shown. Strains were spotted on SC media containing glucose supplemented with or without 0.1 mg/mL of AZC and grown at 30 °C.

Since L-A pathogenesis was correlated with proteostatic defects, we hypothesized that Hsf1 would function as an antiviral factor. Deletion of *HSF1* is lethal, so we utilized the temperature-sensitive allele *hsf1-848* sourced from a previously published collection of strains (61). The *hsf1-848* allele exhibited an absence of growth at 39 °C, an intermediate growth phenotype at 37 °C, and no apparent growth defect at 35 °C (Fig. 4*A*). Spot test assays showed that the *hsf1-848* growth phenotypes at 35 °C and 37 °C were greatly enhanced when combined with either *nuc1∆* or *ski3∆* and that these growth defects were reversed in strains lacking the L-A virus (Fig. 4*A*). As expected, the inviability of all *hsf1-848* mutant strains persisted in cells grown at 39 °C regardless of the presence of L-A. Moreover, using tetrad dissections, we showed that *hsf1-848 nuc1∆ ski3∆* triple mutants were inviable at the permissive temperature if they were infected with L-A but healthy if they were derived from an L-A^0^ strain (*SI Appendix*, Fig. S6). Using western blotting, we found that *hsf1-848 nuc1∆* and *hsf1-848 ski3∆* accumulated increased amounts of L-A Gag compared to the single mutants (Fig. 4*B*). Together with our cell biological studies, these findings suggest that the Hsf1-regulated proteostatic stress response functions as an antiviral system in yeast, opposing the pathogenic consequences of rampant L-A replication.

As proteostatic defects are known to be exacerbated and lead to cytotoxicity at high temperature (59), a simple model attributes the lethal consequences of L-A pathogenesis at high temperature to catastrophic proteostatic stress. To further test this model, we treated strains with azetidine-2-carboxylic acid (AZC), a proline analog that is incorporated into proteins leading to proteostatic stress (38). These experiments showed that *nuc1∆ ski3∆* exhibited strong sensitivity to AZC in a manner dependent on the L-A virus (Fig. 4*C* and *SI Appendix*, Fig. S6). Further, we found that *nuc1∆ ski3∆* exhibited sensitivity to 5% ethanol, a condition that also causes proteostatic defects, but not to 0.5 M NaCl, which causes osmotic stress (*SI Appendix*, Fig. S6). These findings suggest that the lethal consequences of L-A pathogenesis are specifically due to overwhelming proteostatic stress.

## Discussion

Despite its ubiquitous presence in laboratory strains, studies of the L-A dsRNA virus have been limited due to its apparently benign nature. Here, we show that L-A has profound consequences for yeast when its replication is uncontrolled and that diverse innate immune systems maintain L-A replication at a tolerable level. Specifically, we show that, in strains lacking the parallel-acting *NUC1* and *SKI3* antiviral genes, L-A replication is massively upregulated, leading to proteostatic stress and conditional lethality at high temperature. Leveraging this new discovery, we used bioinformatic and forward genetic screens to identify new yeast genes that function to restrict L-A replication or protect cells from the pathogenic consequences of unrestrained L-A replication. As these screens were not saturating, the yeast genome likely encodes numerous other antiviral factors. Many insightful studies have been performed in yeast studying the replication of exogenously introduced viral RNAs from other organisms and it will be interesting to determine if the L-A antiviral factors act similarly on these viral RNAs (62, 63).

Given the clear risk of L-A infection, how it nevertheless persists in the face of ever-present antiviral activity is puzzling. An explanation for this paradox may be that L-A provides a counterbalancing benefit. One possible benefit of L-A is that it enables some strains to maintain satellite viruses that encode secreted toxins that kill neighboring uninfected cells. However, L-A is present in many strains that lack “Killer” satellites, so this explanation is insufficient to explain the persistence of L-A infection. We thus speculate that L-A may have some cryptic benefit that counterbalances its deleterious potential.

Our discovery of Rex2 as a viral attenuation factor expands the arsenal of known mitochondrial antiviral factors beyond Nuc1 and suggests that mitochondria are a key antiviral hub in yeast. Indeed, mitochondria serve central roles in viral defense as a programmed cell death regulator and as a platform for antiviral signaling in humans. How do mitochondrial nucleases attenuate a virus that resides in the cytosol in yeast? One possibility is that these enzymes, while targeted to mitochondria, may nevertheless accumulate to low but sufficient levels in the cytosol to accomplish L-A attenuation directly. Consistent with this hypothesis, we showed previously that Nuc1 accumulates to in the cytosol of meiotic cells, though our methods could not detect it in the cytosol of mitotic cells (27). Another hypothesis is that some aspect of the L-A replication cycle occurs in intimate association with mitochondria. For instance, L-A transcripts may associate with and possibly traverse the mitochondrial, exposing them to Nuc1 and/or Rex2. Our results highlight a potential general importance of mitochondria for viral innate immunity in eukaryotes and position the yeast-L-A system as a powerful model for further studies of this topic.

The antiviral *SKI* complex associates with translating ribosomes and our identification of Pab1, Sro9, and Slf1 as high copy suppressors of L-A pathogenesis further reveal the translating ribosome as a key hub of yeast antiviral activity. The finding that *PAB1* (polyA binding protein) represses L-A is surprising given the absence of polyA tails in L-A transcripts, suggesting that Pab1 does not act on L-A directly. Previous findings showed that L-A transcripts compete with polyA+ yeast mRNAs for capture of 60S ribosomal subunits to form translating 80S complexes (64). One model explaining our findings is that Pab1 enhances the translation of polyA tail-containing mRNAs, which then deplete the availability of 60S subunits for L-A transcripts for translation. The roles of Sro9 and Slf1 in translation are less well-understood, but their functions may similarly relate to competition of L-A transcripts for 60S subunits. Importantly, the homologs of Pab1 and Sro9/Slf1 are involved in human viral defense and further studies of these genes in yeast will shed light on antiviral mechanisms conserved from yeast to human.

We identified an antiviral role for the conserved transcription factor *HSF1* along with an L-A–induced proteostatic stress response involving the accumulation of Hsp104-GFP foci, a *HSF1*-activated marker of cytotoxic protein aggregates. These results support the model in which L-A pathogenesis is caused by proteotoxic stress. We also discovered antiviral function for the SAGA complex, which has been shown to act as a coactivator of Hsf1 target gene induction following heat shock (65, 66). These observations suggest that high levels of L-A lead to the SAGA-dependent activation of Hsf1 target genes which then carry out the antiviral function, illuminating a potential antiviral gene expression program in budding yeast. This model makes many testable predictions that may be relevant to viral pathogenesis in other organisms. Indeed, human *HSF1* also controls the expression of proteostatic regulatory factors. While antiviral functions of human HSF1 have been described, it is unclear what role the proteostatic stress response plays in this (39). Our findings illuminate a powerful system to discern Hsf1’s antiviral function with respect to its role in activation of the proteostatic stress response.

## Method Details

### Strains, Media, and Plasmids.

Standard *S. cerevisiae* genetic and strain manipulation techniques were used for strain construction. Strains were grown at 30 °C in either YPAD or synthetic complete media with appropriate amino acid drop-out for plasmid maintenance unless otherwise specified. Refer to *SI Appendix*, Table S2 for strains and plasmids used in this paper. The yOB255 *URA3::HSE-EmGFP* strain was obtained from Onn Brandman (67). The p5476 *2μ LEU2*, p5476 *NUC1 2μ LEU2*, p5476 *XRN1 2μ LEU2*, p5476 *PAB1 2μ LEU2*, p5476 *SRO9 2μ LEU2*, and p5476 *SLF1 2μ LEU2* MOBY plasmids were obtained from Brenda Andrews (68). The pVB3011 *L-BC GAG (P_GAL1_)* plasmid was obtained from Suzanne Sandmeyer (69). The RLY8470 *trp::mCherry-FIS1TM-KanMX; HSP104-GFP-HIS3* strain was obtained from Rong Li (70).

### Spot analysis.

Yeast strains were grown overnight at 30 °C to saturation. Each strain was diluted to an optical density per mL (OD_600_/mL) of 0.4, serially diluted 10-fold four times, and spotted onto agar plates containing synthetic complete media or drop-out media supplemented with the indicated carbon source (2% glucose or 3% glycerol).

### Protein Extraction and Western Blot.

First, 5 ODs of log-phase cells were harvested and permeabilized with 0.1N NaOH at room temperature for 5 min. The cells were then pelleted and resuspended in SDS/PAGE buffer before heating at 100 °C for 10 min. The samples were centrifuged to isolate the soluble fraction for western blotting. Protein concentrations were determined with an RC/DC assay (BioRad 5000121). Equal amounts of protein were electrophoresed on 10% SDS-PAGE gels and transferred to polyvinylidene difluoride (PVDF) membranes. Membranes were incubated in primary antibody at 4°C overnight and probed with 1:3,000 horseradish peroxidase (HRP)-conjugated horse anti-mouse (7075; Cell Signaling Technology) or goat anti-rabbit (7074; Cell Signaling Technology) secondary antibody. The proteins were detected with Luminata Forte Western HRP Substrate (EMD Millipore) or SuperSignal^TM^ West Atto Ultimate Sensitivity Substrate (A38555, Thermo Scientific) and imaged with the Bio-Rad ChemiDoc XRS+ system. Images were processed with the Image Lab software package (Bio-Rad). The primary antibodies and their dilutions were 1:5,000 anti-Pgk1 (ab113687; Abcam), 1:2,000 anti-L-A Gag (obtained from Reed Wickner), 1:1,000 anti-Sro9, and 1:1,000 anti-Slf1 (obtained from Sandra Wolin) (52).

### Virus Curing.

L-A^0^ strains were generated similar to as we described previously (27). Briefly, a progenitor L-A^0^ strain was isolated following treatment with 32 mM Anisomycin (BioShip ANS245) for 4 d in YPD media and plating for single colonies. Isolates were tested for the presence of the L-A genome by RT-PCR. This L-A^0^ strain was then used for in subsequent backcrosses with *mak3Δ* mutants to generate other L-A^0^ strains.

To evict L-BC, cells were transformed with the *L-BC GAG* (*PGAL1*) plasmid and grown overnight in –URA drop-out media supplemented with 2% raffinose. The saturated culture was diluted and grown in raffinose to log phase before being treated with 2% galactose for 24 h. The resulting culture was plated for single colonies on an agar plate containing synthetic complete media supplemented with 5-FOA. Isolates were tested for the presence of the L-BC genome by RT-PCR.

### RNA Extraction.

First, 10 ODs of log phase cells were harvested and resuspended in phenol solution (P4682; Sigma-Aldrich), SDS, and buffer AE (10 mM Tris·Cl and 0.5 mM EDTA, pH 9.0). Samples were incubated at 65 °C for 30 min and the phase-separated supernatant was washed with chloroform and precipitated in isopropanol overnight. The precipitate was washed with 70% ethanol and dissolved in water. RNA samples were then purified with the RNeasy Mini Kit (Qiagen).

### RT-qPCR.

RT-PCR was performed according to previously described methods (27). Briefly, 900 nanograms of RNA were reverse-transcribed using random nonamers and Maxima H Minus Reverse Transcriptase (Thermo Fisher). The cDNA product was isolated by alkaline hydrolysis and treated with RNase A. Subsequently, qPCR was performed on 1/20 dilutions of cDNA product with the SensiFAST SYBR Hi-ROX Kit (BIO-92005; Meridian Bioscience) on the CFX384 platform (BioRad). The data were plotted using R studio ggplot2.

### Immunofluorescence.

Log phase cells were fixed with 4% formaldehyde and washed two times with 1× phosphate-buffered saline (PBS). The cells were resuspended in sorbitol buffer (1.2 M sorbitol and 20 mM potassium phosphate buffer, pH 7.2) and spheroplated with β-mercaptoethanol (M7522; Sigma-Aldrich) and zymolase at 30°C. Spheroplasted cells were added onto polylysine coated slides and incubated with primary antibody 1:1,000 J2 anti-dsRNA (SCICONS) overnight. The cells were detected with 1:1,000 cyanine3 conjugated goat anti-mouse (Jackson Immuno Research Laboratories) and stained with DAPI. The images were acquired on the Zeiss Axio Imager Z1 with Volocity and processed with ImageJ.

### Growth Curve.

Saturated cultures were diluted to 0.1 OD/mL in YPAD and incubated at 30 °C in the S&P Growth curve robot. Plates were shaken, and the optical density readings were taken every 15 min for 24 h. The data were plotted using R studio ggplot2, and the growth rate was calculated using R studio growthcurver.

### Flow Cytometry.

Log-phase cells were stained with propidium iodide, fixed with 4% formaldehyde, and washed three times with 1× PBS. Fluorescence intensity was measured with Becton Dickinson LSR II flow cytometer and analyzed with Flowing Software. The median GFP intensities of three biological replicates were plotted using R studio ggplot2.

### Fluorescence Microscopy.

Log phase cells were fixed with 4% formaldehyde and washed three times with 1× PBS. Fixed cells were mounted onto microscopy slide and stained with DAPI. Images were acquired with Leica Sp8 confocal LSM and processed with Leica Application Suite (LAS X). The deconvolution program Lightning was applied to all images.

### High Copy Suppression Screen.

A plasmid library of random genomic inserts in the YEP24 plasmid backbone was transformed into a *nuc1Δ ski3Δ* strain and grown on -URA agar plates at high density. Lawns of transformants grown at 30 °C were replica plated to 37 °C and potential suppressors were obtained from the colonies that grew at this temperature. To confirm that the suppression was plasmid dependent, the potential suppressors were first grown on agar plate containing 0.1% 5-fluoroorotic acid (5-FOA) for counterselection and then transferred to a fresh agar plate at 37 °C supplemented with glycerol. The plasmids of the candidates were isolated and sequenced to identify potential genes responsible for the suppression of L-A pathogenesis when overexpressed. The candidate genes were confirmed by spot analysis using p5476 MOBY plasmids.

## Quantification and Statistical Analysis.

Data were tested for statistical significance in Excel and plotted with R studio ggplot2. The details of the statistical tests are described in the figure legends.

## Supplementary Material

Appendix 01 (PDF)

## Data Availability

All study data is available in the main text or *SI Appendix*.
