# Supplementary material for: Diverse yeast antiviral systems prevent lethal pathogenesis caused by the L-A mycovirus
Source: Proc Natl Acad Sci U S A. 2023 Mar 8;120(11):e2208695120. doi: 10.1073/pnas.2208695120 (PMC10089162; doi:10.1073/pnas.2208695120)
Supplement: Supplementary file 1 — Appendix 01 (PDF) [file pnas.2208695120.sapp.pdf]

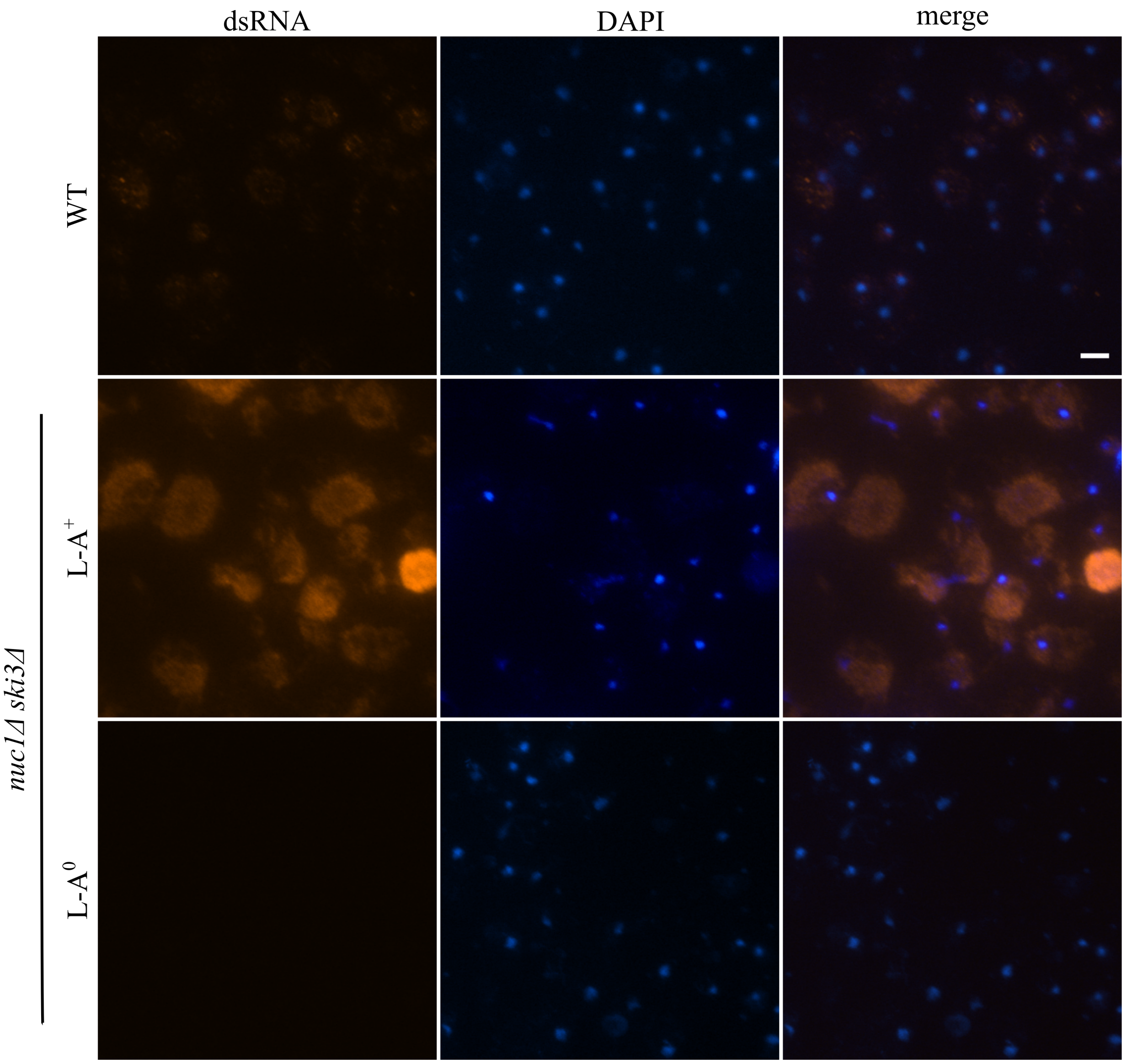

Figure S1. Additional images of immunofluorescence of dsRNA (red) of strains from Figure 1B. Images of fields of cells of indicated genotype is shown. Cells are stained with DAPI to visualize nuclei. Scale bar, 30 μm.

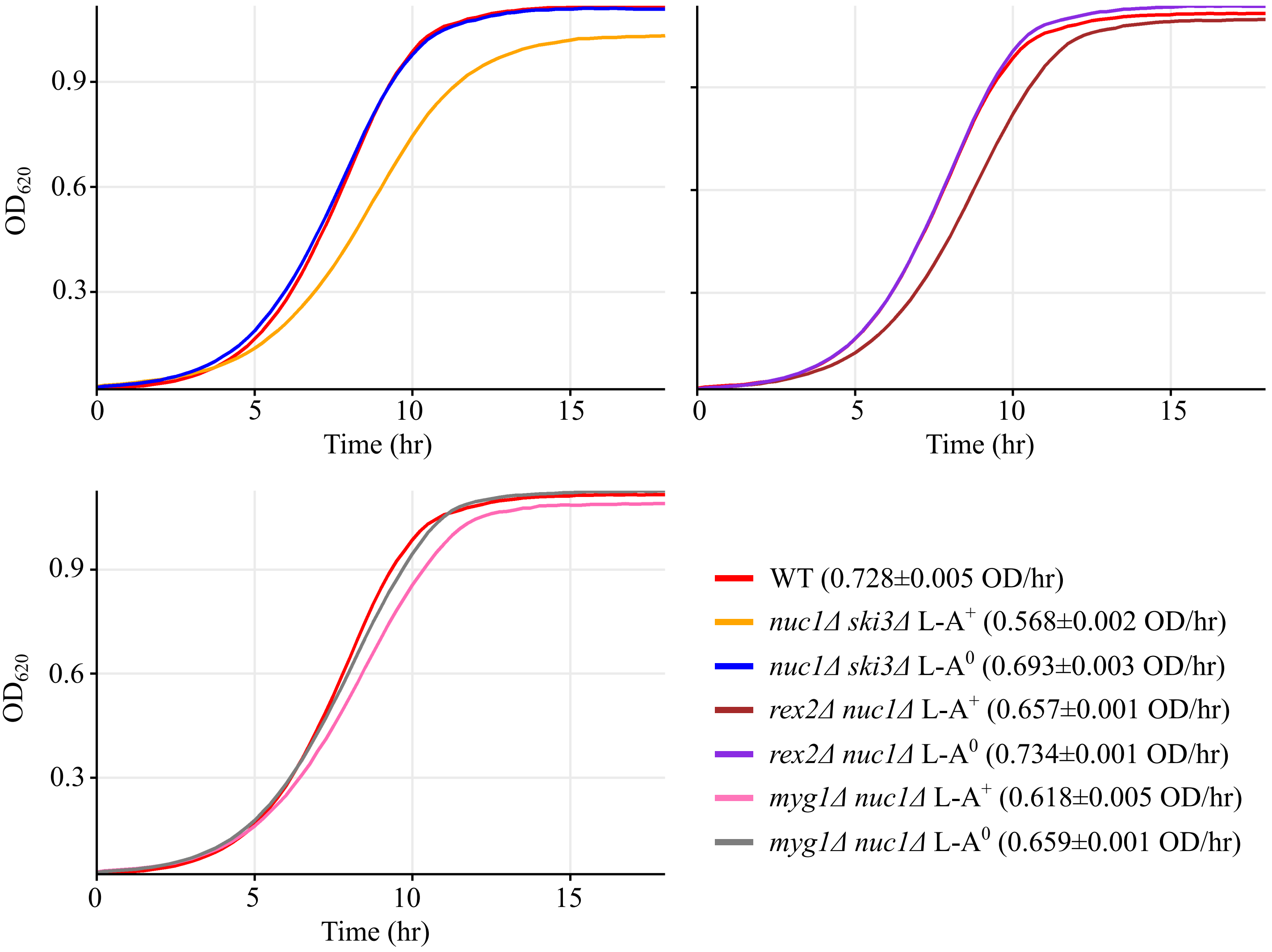

Figure S2. Quantitative measurement of L-A pathogenesis. Cells were grown at 30°C for 18 hours. Density measurements of cell cultures were taken every 15 minutes. The average maximum growth rate and standard deviation of each genotype is reported. n = 4. The growth rate was calculated using Growthcurver in R.

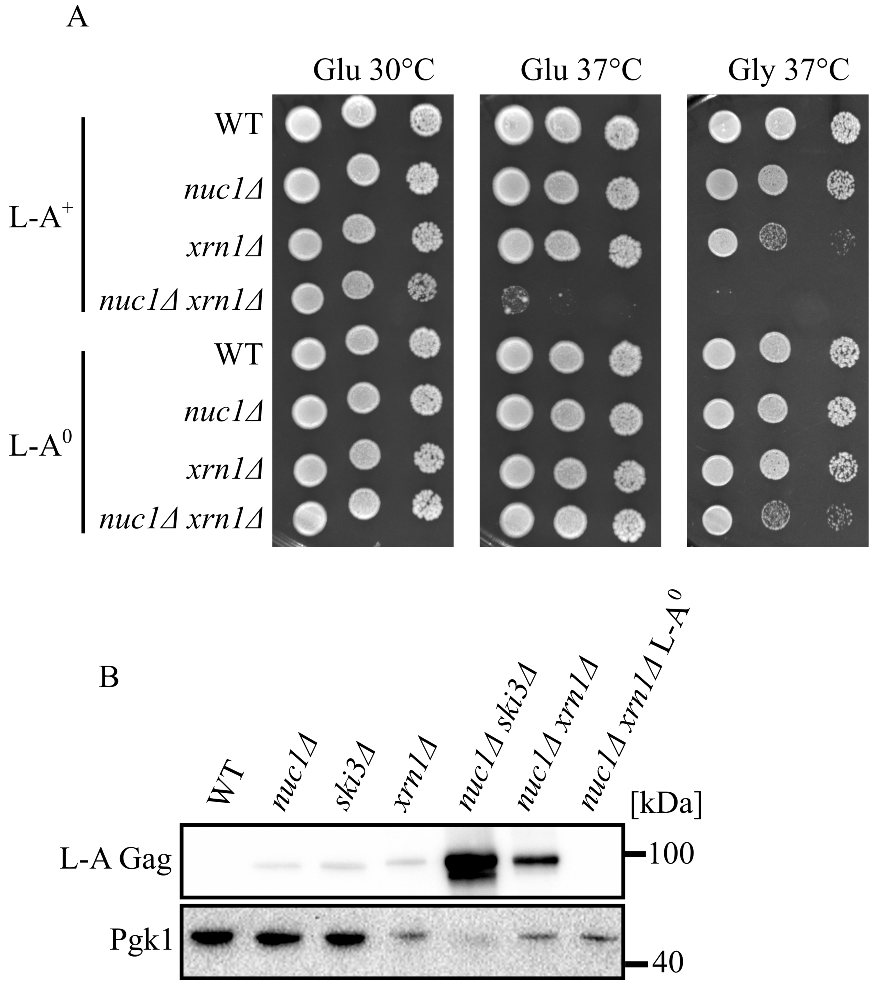

Figure S3. *XRN1*, *NUC1*, and *SKI3* work in parallel pathways to regulate L-A Gag level. **(A)** Spot analysis of strains defective in *NUC1* and *XRN1* is shown. Strains are spotted on SC media containing either glucose or glycerol and grown at the indicated temperature. **(B)** Western blotting of L-A Gag and Pgk1 protein levels of strains in Figure S2A. Molecular weight markers are indicated on the right.

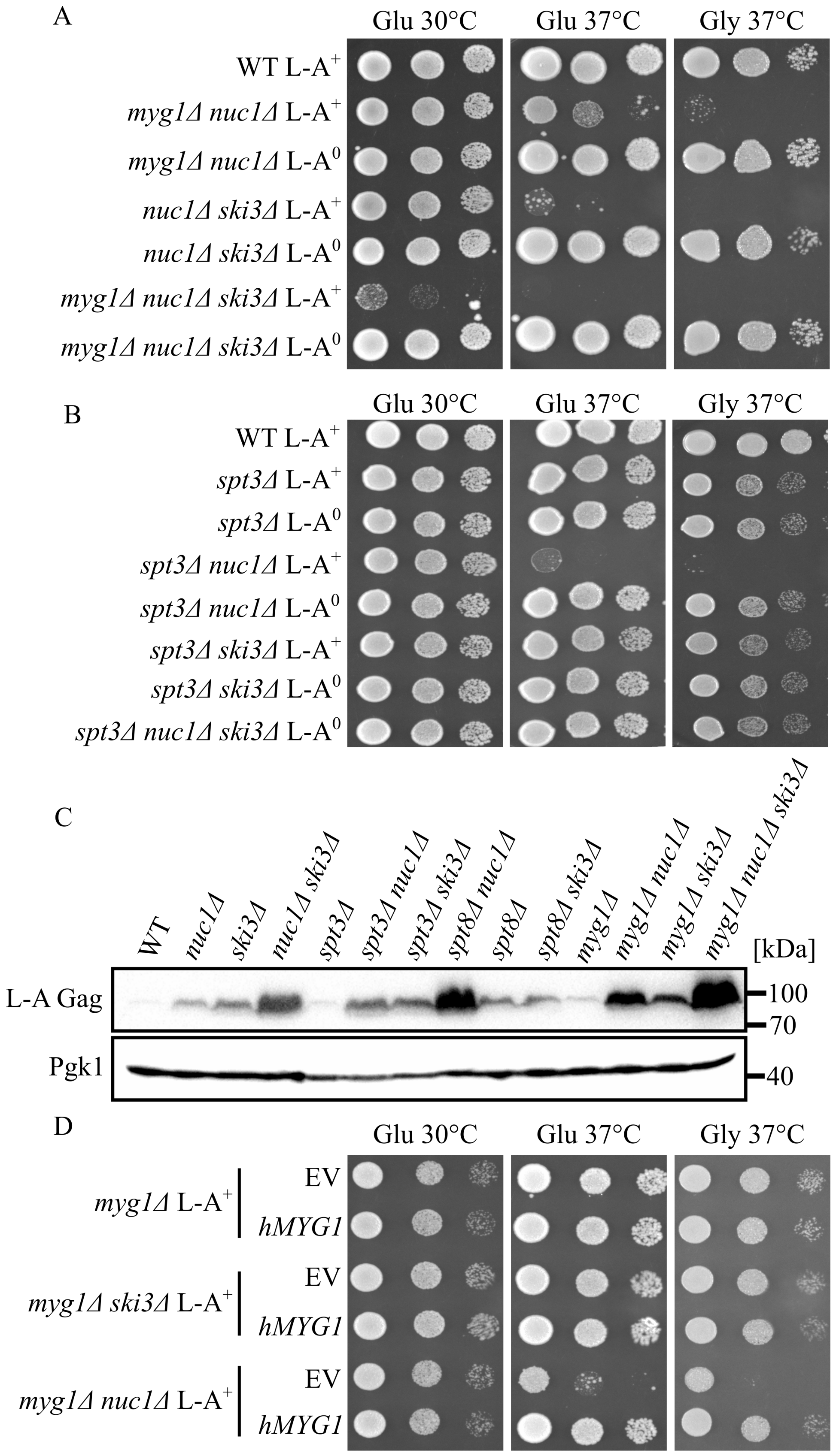

Figure S4. Additional new antiviral factors *MYG1* and *SPT3* contribute to L-A pathogenesis. **(A)** Spot analysis of strains defective in *MYG1*, *NUC1*, and *SKI3* with and without L-A is shown. Strains are spotted on SC media containing either glucose or glycerol and grown at the indicated temperature. **(B)** Spot analysis of strains defective in *SPT3*, *NUC1*, and *SKI3* with and without L-A is shown. Strains are spotted on SC media containing either glucose or glycerol and grown at the indicated temperature. **(C)** Western blotting detection of L-A Gag and Pgk1 protein levels in the indicated strains is shown. Molecular weight markers are indicated on the right. **(D)** Spot analysis of strains from S4A expressing *hMYG1* on a plasmid. Strains are spotted on -URA media containing either glucose or glycerol and grown at the indicated temperatures.

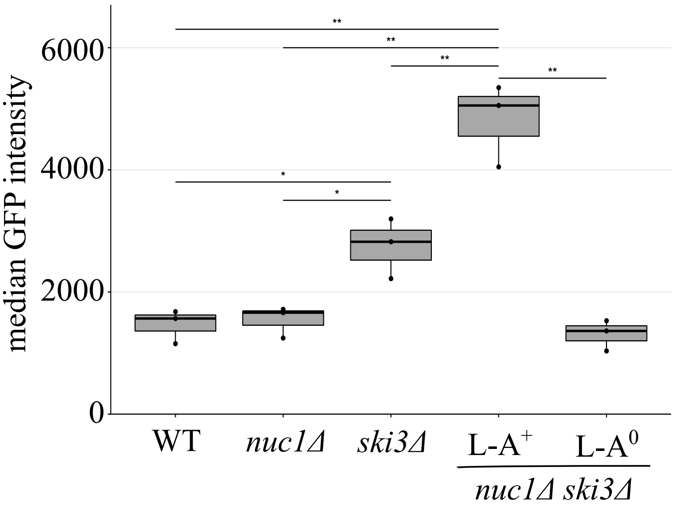

Figure S5. L-A pathogenesis correlates with proteostatic stress. Flow cytometry was used to measure HSE-GFP expression in the indicated strains. Median GFP intensity is shown. n = 3. * $p < 0.05$, ** $p < 0.01$. The *p* value was calculated using unpaired student's t-test.

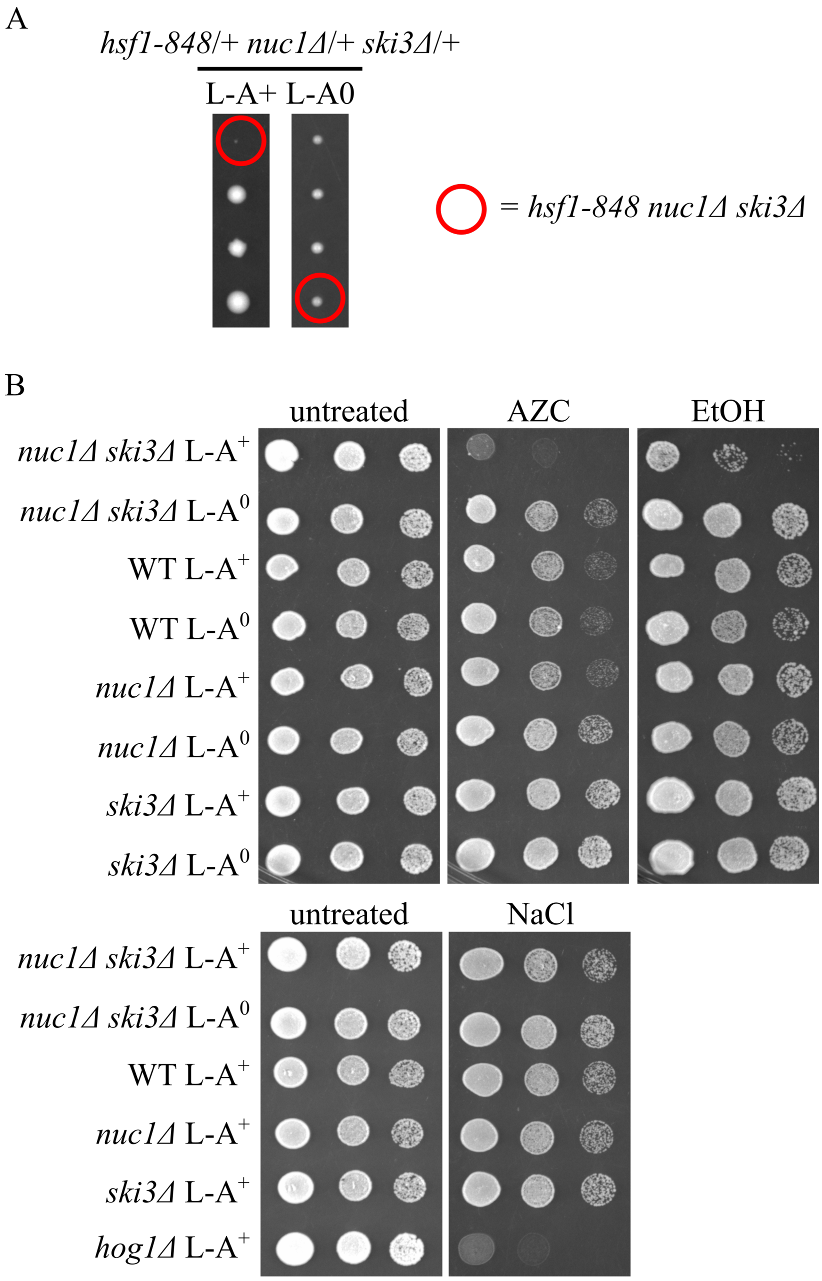

Figure S6. High L-A viral level sensitizes cells to proteostatic stress. **(A)** Tetrad dissections of L-A$^+$ (left) and L-A^0 (right) *hsf1-848*/+ *nuc1Δ*/+ *ski3Δ*/+ mutants. Known or inferred genotype of interest (*hsf1-848 nuc1Δ ski3Δ*) are indicated in red. **(B)** Spot analysis of strains treated with various types of stressors is shown. Strains were spotted on SC media containing glucose supplemented with 0.1 mg/mL of AZC [proteostatic stress], 5% ethanol (EtOH) [proteostatic stress], 0.5M of sodium chloride (NaCl) [osmotic stress], or 200 J/m^2 UVC [DNA damage stress].

Table S1: Strain and plasmid table

Identification	Relevant Genotype	Plasmid
jgy33	BY4741 *MAT*a L-A^0	
jgy36	BY4741 *MAT*a L-A^0 *nuc1Δ::NatMX6*	
jgy38	BY4741 *MAT*a L-A^0 *ski3Δ::HygMX6*	
jgy42	BY4741 *MAT*a L-A^0 *nuc1Δ::NatMX6 ski3Δ::HygMX6*	
jgy45	BY4741 *MAT*a L-A$^+$ *nuc1Δ::NatMX6*	
jgy47	BY4741 *MAT*a L-A$^+$ *ski3Δ::HygMX6*	
jgy49	BY4741 *MAT*a L-A$^+$ *nuc1Δ::NatMX6 ski3Δ::HygMX6*	
jgy156	BY4741 *MAT*a L-A$^+$	p5476 *2μ LEU2*
jgy157	BY4741 *MAT*a L-A$^+$ *nuc1Δ::NatMX6 ski3Δ::HygMX6*	p5476 *2μ LEU2*
jgy158	BY4741 *MAT*a L-A$^+$ *nuc1Δ::NatMX6 ski3Δ::HygMX6*	p5476 *PAB1 2μ LEU2*
jgy171	BY4741 *MAT*a L-A$^+$ *nuc1Δ::NatMX6 ski3Δ::HygMX6*	p5476 *SRO9 2μ LEU2*
jgy173	BY4741 *MAT*a L-A$^+$ *nuc1Δ::NatMX6 ski3Δ::HygMX6*	p5476 *SLF1 2μ LEU2*
jgy225	BY4741 *MAT*a L-A$^+$	
mmy2334	BY4741 *MAT*a L-A$^+$ *hog1Δ::KanMX6*	
mmy4076	BY4741 *MAT*α L-A$^+$ *nuc1Δ::NatMX6*	
mmy4699	BY4741 *MAT*α L-A$^+$	
mmy6042	BY4741 *MAT*a L-A$^+$	
mmy6860	BY4741 *MAT*a L-A$^+$ *ski3Δ::HygMX6*	
mmy7701	BY4741 *MAT*a L-A$^+$	
mmy8064	BY4741 *MAT*α L-A^0	
mmy8213	BY4741 *MAT*a L-A$^+$ *nuc1Δ::NatMX6 ski3Δ::HygMX6*	
mmy8244	BY4741 *MAT*α L-A^0	
mmy8245	BY4741 *MAT*a L-A^0	
mmy8246	BY4741 *MAT*α L-A^0 *nuc1Δ::NatMX6*	
mmy8247	BY4741 *MAT*a L-A^0 *nuc1Δ::NatMX6*	
mmy8466	BY4741 *MAT*a L-A^0 *nuc1Δ::NatMX6*	
mmy9292	BY4741 *MAT*α L-A$^+$ *nuc1Δ::NatMX6 ski3Δ::HygMX6*	p5476 *2μ LEU2*
mmy9293	BY4741 *MAT*α L-A$^+$ *nuc1Δ::NatMX6 ski3Δ::HygMX6*	p5476 *NUC1 2μ LEU2*
mmy9320	BY4741 *MAT*α L-A$^+$ *nuc1Δ::NatMX6*	p5476 *2μ LEU2*
mmy9321	BY4741 *MAT*α L-A$^+$	p5476 *2μ LEU2*
mmy9322	BY4741 *MAT*α L-A^0 *nuc1Δ::NatMX6 ski3Δ::HygMX6*	p5476 *2μ LEU2*
mmy9323	BY4741 *MAT*α L-A$^+$ *ski3Δ::HygMX6*	p5476 *2μ LEU2*

mmy9342	BY4741 *MAT*a L-A^{+} *nuc1Δ::NatMX6 rex2Δ::KanMX6*	
mmy9344	BY4741 *MAT*a L-A^{+} *rex2Δ::KanMX6*	
mmy9350	BY4741 *MAT*α L-A^{+} *nuc1Δ::NatMX6 ski3Δ::HygMX6*	p5476 *XRN1 2μ LEU2*
mmy9351	BY4741 *MAT*α L-A^{+} *nuc1Δ::NatMX6 xrn1Δ::KanMX6*	
mmy9353	BY4741 *MAT*α L-A^{+} *xrn1Δ::KanMX6*	
mmy9609	BY4741 *MAT*α L-A^{0} *xrn1Δ::NatMX6*	
mmy9611	BY4741 *MAT*α L-A^{0} *xrn1Δ::NatMX6 nuc1Δ::HygMX6*	
mmy10001	BY4741 *MAT*a L-A^{+} *nuc1Δ::NatMX6 rex2Δ::HygMX6 xrn1Δ::KanMX6*	pRS316 *NUC1-FLAG URA3*
mmy10003	BY4741 *MAT*α L-A^{0} *xrn1Δ::NatMX6 nuc1Δ::HygMX6 rex2Δ::KanMX6*	pRS316 *NUC1-FLAG URA3*
mmy10007	BY4741 *MAT*α L-A^{0} *URA3::HSE-EmGFP nuc1Δ::NatMX6 ski3Δ::HygMX6*	
mmy10073	BY4741 *MATα* L-A^{+} *URA3::HSE-EmGFP nuc1Δ::NatMX6*	
mmy10074	BY4741 *MAT*α L-A^{+} *URA3::HSE-EmGFP nuc1Δ::NatMX6 ski3Δ::HygMX6*	
mmy10076	BY4741 *MAT*a L-A^{+} *URA3::HSE-EmGFP ski3Δ::HygMX6*	
mmy10077	BY4741 *MAT*α L-A^{+} *URA3::HSE-EmGFP*	
mmy10215	BY4741 *MAT*a L-A^{+} *spt3Δ::KanMX6*	
mmy10216	BY4741 *MAT*a L-A^{+} *nuc1Δ::NatMX6 spt3Δ::KanMX6*	
mmy10217	BY4741 *MAT*a L-A^{+} *ski3Δ::HygMX6 spt3Δ::KanMX6*	
mmy10258	BY4741 *MAT*α L-A^{+}	
mmy10275	BY4741 *MAT*a L-A^{+} *spt8Δ::KanMX6*	
mmy10276	BY4741 *MAT*a L-A^{+} *ski3Δ::HygMX6 spt8Δ::KanMX6*	
mmy10277	BY4741 *MAT*a L-A^{+} *nuc1Δ::NatMX6 spt8Δ::KanMX6*	
mmy10342	BY4741 *MAT*a L-A^{0} *spt3Δ::KanMX6*	
mmy10343	BY4741 *MAT*a L-A^{0} *nuc1Δ::NatMX6 spt3Δ::KanMX6*	
mmy10344	BY4741 *MAT*a L-A^{0} *ski3Δ::HygMX6 spt3Δ::KanMX6*	
mmy10345	BY4741 *MAT*a L-A^{0} *nuc1Δ::NatMX6 ski3Δ::HygMX6 spt3Δ::KanMX6*	
mmy10461	BY4741 *MAT*a L-A^{+} *nuc1Δ::NatMX6 ski3Δ::HygMX6*	p5476 *2μ LEU2*

mmy10462	BY4741 *MAT*a L-A$^+$ *nuc1Δ::NatMX6 ski3Δ::HygMX6*	p5476 *SRO9 2μ LEU2*
mmy10463	BY4741 *MAT*a L-A$^+$ *nuc1Δ::NatMX6 ski3Δ::HygMX6*	p5476 *SLF1 2μ LEU2*
mmy10464	BY4741 *MAT*a L-A$^+$ *nuc1Δ::NatMX6 ski3Δ::HygMX6*	p5476 *PAB1 2μ LEU2*
mmy10845	BY4741 *MAT*α L-A$^+$ *nuc1Δ::NatMX6 myg1Δ::KanMX6*	
mmy10846	BY4741 *MAT*a L-A$^+$ *ski3Δ::HygMX6 myg1Δ::KanMX6*	
mmy10847	BY4741 *MAT*α L-A$^+$ *myg1Δ::KanMX6*	
mmy10848	BY4741 *MAT*α L-A^0 *nuc1Δ::NatMX6 ski3Δ::HygMX6 myg1Δ::KanMX6*	
mmy10849	BY4741 *MAT*a L-A^0 *nuc1Δ::NatMX6 myg1Δ::KanMX6*	
mmy10852	BY4741 *MAT*α L-A$^+$ *nuc1Δ::NatMX6 ski3Δ::HygMX6 myg1Δ::KanMX6*	
scy629	BY4741 *MAT*a L-A^0 *rex2Δ::KanMX6*	
scy630	BY4741 *MAT*a L-A^0 *nuc1Δ::NatMX6 rex2Δ::KanMX6*	
scy633	BY4741 *MAT*a L-A$^+$ *nuc1Δ::NatMX6 ski3Δ::HygMX6 rex2Δ::KanMX6*	pRS316 *NUC1 URA3*
scy635	BY4741 *MAT*a L-A^0 *nuc1Δ::NatMX6 ski3Δ::HygMX6 rex2Δ::KanMX6*	pRS316 *NUC1 URA3*
scy838	BY4741 *MAT*a L-A$^+$ L-BC^0 *nuc1Δ::NatMX6 ski3Δ::HygMX6*	
scy839	BY4741 *MAT*a L-A$^+$ L-BC^0	
scy841	BY4741 *MAT*a L-A$^+$ L-BC^0 *ski3Δ::HygMX6*	
scy842	BY4741 *MAT*a L-A$^+$ L-BC^0 *nuc1Δ::NatMX6*	
scy894	BY4741 *MAT*a L-A^0 L-BC^0 *nuc1Δ::NatMX6 ski3Δ::HygMX6*	
scy1100	BY4741 *MAT*a L-A$^+$ *URA3::HSE-EmGFP*	p5476 *2μ LEU2*
scy1104	BY4741 *MAT*α L-A$^+$ *URA3::HSE-EmGFP nuc1Δ::NatMX6 ski3Δ::HygMX6*	p5476 *2μ LEU2*
scy1105	BY4741 *MAT*α L-A$^+$ *URA3::HSE-EmGFP nuc1Δ::NatMX6 ski3Δ::HygMX6*	p5476 *PAB1 2μ LEU2*
scy1106	BY4741 *MAT*α L-A$^+$ *URA3::HSE-EmGFP nuc1Δ::NatMX6 ski3Δ::HygMX6*	p5476 *SLF1 2μ LEU2*
scy1107	BY4741 *MAT*α L-A$^+$ *URA3::HSE-EmGFP nuc1Δ::NatMX6 ski3Δ::HygMX6*	p5476 *SRO9 2μ LEU2*
scy1194	BY4741 *MAT*a L-A$^+$ *trp::mCherry-FIS1TM-URA3 HSP104-GFP::HIS3 nuc1Δ::NatMX6*	
scy1195	BY4741 *MAT*a L-A$^+$ *trp::mCherry-FIS1TM-URA3 HSP104-GFP::HIS3 ski3Δ::HygMX6*	

scy1233	BY4741 *MAT*a L-A$^+$ *trp::mCherry-FIS1TM-URA3 HSP104-GFP::HIS3*	
scy1240	BY4741 *MAT*a L-A$^+$ *trp::mCherry-FIS1TM-URA3 HSP104-GFP::HIS3 nuc1Δ::NatMX6 ski3Δ::HygMX6*	
scy1279	BY4741 *MAT*α L-A$^+$ *nuc1 Δ::NatMX6 myg1Δ::KanMX6*	P426 *GPD 2μ URA3*
scy1280	BY4741 *MAT*α L-A$^+$ *ski3Δ::HygMX6 myg1Δ::KanMX6*	P426 *GPD 2μ URA3*
scy1281	BY4741 *MAT*α L-A$^+$ *myg1Δ::KanMX6*	P426 *GPD 2μ URA3*
scy1285	BY4741 *MAT*a L-A^0 *trp::mCherry-FIS1TM-URA3 HSP104-GFP::HIS3 nuc1Δ::NatMX6 ski3Δ::HygMX6*	
scy1288	BY4741 *MAT*α L-A$^+$ *nuc1 Δ::NatMX6 myg1Δ::KanMX6*	P426 *GPD hMYG1 2μ URA3*
scy1289	BY4741 *MAT*α L-A$^+$ *ski3Δ::HygMX6 myg1Δ::KanMX6*	P426 *GPD hMYG1 2μ URA3*
scy1290	BY4741 *MAT*α L-A$^+$ *myg1Δ::KanMX6*	P426 *GPD hMYG1 2μ URA3*

*All strains are congenic to BY4741: *his3-Δ1 leu2-Δ0 met15-Δ0 ura3-Δ0*.
